# Long-Term Survival Outcomes After Minimally Invasive Surgery for Ileal Neuroendocrine Tumors

**DOI:** 10.1245/s10434-024-15468-6

**Published:** 2024-05-26

**Authors:** Akitada Yogo, Alan Paciorek, Yosuke Kasai, Farhana Moon, Kenzo Hirose, Carlos U. Corvera, Emily K. Bergsland, Eric K. Nakakura

**Affiliations:** 1 Division of Surgical Oncology, Section of Hepatopancreaticobiliary Surgery, Department of Surgery, University of California, San Francisco, San Francisco, CA USA; 2 Helen Diller Family Comprehensive Cancer Center, San Francisco, CA USA; 3 Department of Epidemiology and Biostatistics, University of California, San Francisco, San Francisco, CA USA; 4 Department of Surgery, Kyoto University, Kyoto, Japan; 5 Division of Hematology and Oncology, Department of Medicine, University of California, San Francisco, San Francisco, CA USA

**Keywords:** Minimally invasive surgery, Ileal neuroendocrine tumors, Mesenteric mass, Hand-assisted laparoscopic surgery, Propensity score

## Abstract

**Background:**

Ileal neuroendocrine tumors (i-NETs) are characterized by their multifocality and bulky mesenteric mass. Having shown that minimally invasive surgery (MIS) utilizing a hand-access port device has favorable short-term outcomes and achieves the goals of surgery for i-NETs, we sought to analyze long-term survival outcomes of MIS.

**Methods:**

One hundred and sixty-eight patients who underwent resection of primary i-NETs at a single institution between January 2007 and February 2023 were retrospectively studied. Patients were categorized into the MIS or open surgery cohorts on an intention-to-treat basis. Open surgery was selected mainly based on the need for hepatectomy or bulky mesenteric mass resection. Overall survival was analyzed using log-rank tests with propensity score matching (PSM) and Cox proportional hazards regression. PSM was performed to reduce standardized mean differences of the variables to <0.2.

**Results:**

Overall, 129 (77%) patients underwent MIS and 39 (23%) underwent open surgery. Twenty-seven MIS patients were converted to an open procedure. The median follow-up time was 49 months (interquartile range 23–87 months). In the PSM cohorts, overall survival did not differ significantly between the MIS and open surgery cohorts {median 99 months (95% confidence interval [CI] 91–not applicable [NA]) vs. 103 months (95% CI 86–NA), *p* = 0.77; hazard ratio 0.87 (95% CI 0.33–2.2), *p* = 0.77}.

**Conclusions:**

MIS is an alternative to open surgery for i-NETs, achieving similar short- and long-term oncological outcomes. Bulky mesenteric mass and a plan for concurrent liver resection are potential criteria for open surgery.

**Supplementary Information:**

The online version contains supplementary material available at 10.1245/s10434-024-15468-6.

Neuroendocrine tumors (NETs) of the small intestine have an increasing incidence and now account for 37% of all small bowel malignancies in the US, making them the most common small bowel tumor.^1,2^ The most common site is the ileum, and ileal NETS (i-NETS) are characterized by their small size and frequent multifocality.

The North American Neuroendocrine Tumor Society guidelines recommend identifying primary tumors by palpation using the surgeon’s fingers, and state that a pure laparoscopic approach is inadequate.^3^ i-NETS may present as a bulky lymphadenopathy/mesenteric mass, which causes small bowel obstruction and can hinder complete surgical resection when the mass extends to the root of the superior mesenteric vessels. Thus, the European Neuroendocrine Tumor Society does not recommend laparoscopic surgery for cases with large mesenteric infiltration as well as multifocal primary tumors.^4^

Minimally invasive surgery (MIS) uses smaller incisions via laparoscopic or robotic methods and is widely used for gastrointestinal diseases. Although various guidelines mention possible benefits of using MIS for i-NETs, none offer recommendations regarding specific techniques or established criteria for patient selection.^3–12^ Having previously shown that MIS utilizing a hand-access port device had favorable short-term outcomes and achieved the goals of surgery for i-NETs,^9,11^ we sought to analyze long-term survival outcomes of this approach.

## Materials and Methods

### Study Design and Patients

We conducted a single-institution, retrospective cohort study of 168 patients who underwent resection of primary i-NETs between January 2007 and February 2023. A total of 182 i-NET patients were identified, however 14 patients were excluded because preoperative or postoperative imaging was unavailable. The 168 patients were divided into two groups based on the surgical approach (MIS or open) using an intention-to-treat basis; 108 of these were included in the earlier study.^9,11^ This study was performed in accordance with the Declaration of Helsinki, and Health Insurance Portability and Accountability Act (HIPAA) compliance and was approved by the Institutional Review Board at University of California, San Francisco (UCSF). Informed consent was waived because of the retrospective design and the use of anonymous patient data.

### Surgical Approaches and Indications

For each patient, the surgical approach was determined after multidisciplinary discussion. Open surgery was performed for bulky mesenteric mass or concurrent liver resection, except for left lobectomy. Otherwise, MIS was performed as described previously, using a hand-access port (GelPort, Applied Medical, Rancho Santa Margarita, CA, USA).^9^ MIS included conversion from MIS to an open procedure, which was defined as an extension of the main skin incision with removal of the hand-access base retractor for any reason.^13^

### Outcomes and Variables

The primary outcome was overall survival (OS), i.e. the length of time from the surgery date to the patient’s death. We also investigated effect modifiers and mediators of mortality, with various surgical procedures as the exposure. Data were collected from medical records, preoperative and postoperative imaging, operative reports, and pathological reports. Patient demographics, tumor characteristics, and the presence of concurrent liver surgery were considered possible predictors/effect modifiers and potential confounders. Variables related to surgical outcomes, incomplete mesenteric lymph node dissection, postoperative complications, and postoperative treatment were considered as possible mediators. Incomplete mesenteric lymph node dissection (R2) was defined by mention in the operative report or the presence of residual mesenteric mass, which was evaluated by comparing the preoperative and postoperative follow-up imaging. The microscopically positive mesenteric margin (R1) was defined if tumor cells presented within 1 mm from the edge of the surgical specimen of the mesentery, without incomplete mesenteric lymph node dissection (R2). Complete mesenteric lymph node dissection (R0) was defined as neither incomplete mesenteric lymph node dissection (R2) nor microscopically positive mesenteric margin (R1). Postoperative complications were graded using the Clavien–Dindo classification. Liver-directed therapy involved transarterial bland embolization/chemoembolization, Y90 radioembolization, radiation, and microwave ablation. Systemic medications included everolimus, capecitabine, temozolomide, pazopanib, carboplatin/etoposide, gemcitabine/oxaliplatin, and 5-fluorouracil/leucovorin/oxaliplatin.

### Statistical Analysis

Continuous variables are represented as medians with interquartile ranges, and categorical variables are represented as frequencies and percentages. The Wilcoxon rank-sum and Fisher's exact test were used to compare distributions of potential confounders across groups by surgical approach. Survival curves were constructed using the Kaplan–Meier method and compared using the log-rank test. A multivariable Cox proportional hazards regression model was used to calculate for surgical approaches (open surgery, MIS without conversion, and conversion from MIS to open surgery) and other variables the hazard ratio (HR) of overall mortality from the date of resection. Risk factors used in the multivariable analysis were selected based on prior knowledge.^14–17^ Propensity score matching (PSM) was performed to decrease the standardized mean difference (SMD) of as many potential confounders as possible to <0.2,^18,19^ based on the variables of age, history of small bowel obstruction, tumor grade (G1 vs. G2), T staging (T1/2 vs. T3/4), N staging (N− vs. N+), M staging (M0 vs. M1a/M1b/M1c), overall staging (I/II vs. III/IV), the extent of involvement of the mesenteric mass to the root of the mesenteric vessels (region 0/1/2 vs. region 3),^9^ mesenteric mass larger than 2 cm,^17^ and the presence of concurrent liver surgery at a ratio of 1:2 using a 0.1 caliper. All statistical analyses and representations were performed using R (v.4.1.1; The R Foundation for Statistical Computing, Vienna, Austria) with R packages multcomp (v.1.4-25), survival (v.3.5-5), ggplot2 (v.3.4.2), MatchIt (v.4.5.3), tableone (v.0.13.2), and survminer (v.0.4.9).

## Results

### Patient and Tumor Characteristics

Overall, 129 (77%) patients underwent MIS and 39 (23%) underwent open surgery (Table 1, Online Resource Table 1). The MIS cases included 27 surgeries converted to an open procedure due to large lymphadenopathy/mesenteric mass in 17 cases, liver surgery in 4 cases, and other technical difficulties such as organ adhesion in 6 cases. The overall median follow-up time was 49 (interquartile range [IQR] 23–87) months. Between the two cohorts, there was no difference in age, sex, body mass index, existence of carcinoid syndrome, history of small bowel obstruction, or the number of primary tumors (Tables 2 and 3). Tumor grade 2 or 3 was associated with open surgery compared with MIS cases {odds ratio 2.0 (95% confidence interval [CI] 0.87–4.4), *p* = 0.085}. The presence of a mesenteric mass extending to the root of the mesenteric vessels was more common in open surgery (23% vs. 10%, odds ratio 2.7 [95% CI 0.91–7.5], *p* = 0.055) (Table 2). The size of the mesenteric mass was 2.6 (IQR 1.1–3.5) cm in open surgery and 1.8 (IQR 1.0–3.2) cm in MIS surgery (*p* = 0.20), and larger than 2 cm^17^ in 66% and 48% of open surgery and MIS cases, respectively (odds ratio 2.1 [95% CI 0.92–4.8], *p* = 0.065). The mesenteric lymph node/mass dissection was incomplete in 23% of the open surgeries and 14% of the MIS surgeries (*p* = 0.20). At the time of resection, hepatic metastasis was present in 56% and 50% of open surgery and MIS cases, respectively (*p* = 0.20). The types of Frilling classification of liver metastasis^16^ were not different between the cohorts, but concurrent liver surgery was more frequent in open surgery (26% vs. 7%, *p* = 0.003). There was no difference in postoperative treatments (Table 3).

**Table 1:** Demographics and patient backgrounds of the entire cohort

Characteristic	*N*	Surgical procedure		
		Open [*n* = 39]	MIS [*n* = 129]	*p* value
Surgical procedure	168			
Open		39 (100)	0 (0)	
MIS without conversion		0 (0)	102 (79)	
MIS converted to open		0 (0)	27 (21)	
Age, years [median (IQR)]	168	61 (53–67)	60 (53–67)	0.62
Sex, female	168	20 (51)	62 (48)	0.86
BMI, kg/m^2^ [median (IQR)]	167	27.0 (24.2–29)	26.5 (22.6–31)	0.98
Race, White	165	29 (76)	97 (76)	1
Carcinoid syndrome	165	20 (53)	69 (54)	0.86
SSA before resection	168	14 (36)	38 (29)	0.44
History of small bowel obstruction	168	29 (74)	88 (68)	0.55

Data are expressed as *n* (%) unless otherwise specified

*MIS* minimally invasive surgery, *IQR* interquartile range, *SSA* somatostatin analog, *BMI* body mass index

**Table 2:** Tumor characteristics of the entire cohort

Characteristic	*N*	Surgical procedure		
		Open [*n* = 39]	MIS [*n* = 129]	*p* value
Size of primary tumor, cm [median (IQR)]	166	1.9 (1.3–3)	1.9 (1.3–3)	0.91
No. of primary tumor(s) [median (IQR)]	167	1 (1–4)	1 (1–2)	0.37
Multiple tumors	167	17 (45)	49 (38)	0.46
Mesenteric mass involvement to the root of the mesentery	168	9 (23)	13 (10)	0.055
Size of mesenteric mass,cm [median (IQR)]	165	2.6 (1.1–3.5)	1.8 (1.0–3.2)	0.21
Mesenteric mass >2 cm in size	165	25 (66)	61 (48)	0.065
Tumor grade	160			0.14
1		18 (49)	80 (65)	
2		19 (51)	42 (34)	
3		0 (0)	1 (0.8)	
T staging	167			0.35
T1		2 (5.3)	10 (7.8)	
T2		3 (7.9)	24 (19)	
T3		19 (50)	60 (47)	
T4		14 (37)	35 (27)	
N+	166	36 (95)	122 (97)	0.62
M staging	168			0.25
M0		13 (33)	59 (46)	
M1a		13 (33)	45 (35)	
M1b		4 (10)	6 (4.7)	
M1c		9 (23)	19 (15)	
Stage	168			0.23
I		0 (0)	4 (3.1)	
II		1 (2.6)	1 (0.8)	
III		12 (31)	54 (42)	
IV		26 (67)	70 (54)	
Frilling liver metastasis classification	168			0.34
0		17 (44)	65 (50)	
1		1 (2.6)	5 (3.9)	
2		20 (51)	59 (46)	
3		1 (2.6)	0 (0)	
Concurrent liver surgery	168	10 (26)	9 (7.0)	0.0029

Data are expressed as *n* (%) unless otherwise specified

*IQR* interquartile range, *MIS* minimally invasive surgery

**Table 3:** Characteristics related to surgical outcomes of the entire cohort

Characteristic	*N*	Surgical procedure		
		Open [*n* = 39]	MIS [*n* = 129]	*p* value
Incomplete mesenteric lymph node dissection	167	9 (23)	18 (14)	0.21
Microscopically positive mesenteric margin	168	7 (18)	20 (16)	0.80
Complete mesenteric lymph node dissection	167	23 (59)	90 (70)	0.24
Estimated blood loss, mL [median (IQR)]	161	100 (30–150)	50 (20–100)	0.0082
Postoperative complications Grade ≥3	168	4 (10)	4 (3.1)	0.085
Length of stay, days [median (IQR)]	168	6 (5–7)	5 (4–7)	0.063
Postoperative treatment	168			
SSA or telotristat		19 (49)	61 (47)	1
Liver-directed therapy		10 (26)	30 (23)	0.83
Surgical resection		5 (13)	13 (10)	0.57
PRRT		6 (15)	19 (15)	1
Systemic medication		8 (21)	14 (11)	0.17
Survival status dead	168	10 (26)	34 (27)	1
Follow-up months [median (IQR)]	168	46 (16–99)	50 (25–83)	0.62

Data are expressed as *n* (%) unless otherwise specified

*MIS* minimally invasive surgery, *IQR* interquartile range, *SSA* somatostatin analog, *PRRT* peptide receptor radionuclide therapy

### Survival Analysis Using Propensity Score Matching

**Fig. 1:**
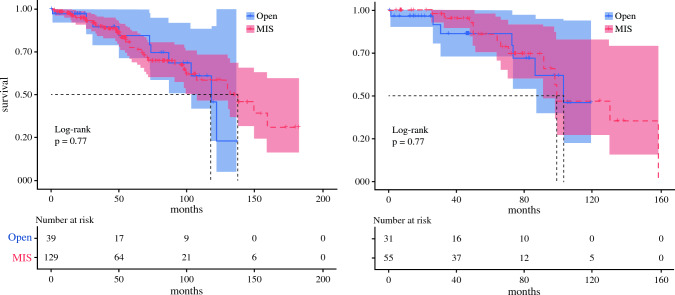
Survival curves. Kaplan–Meier overall survival curves are shown for the entire cohort (left) and the propensity score matched cohorts (right). Shaded areas are 95% confidence intervals. *MIS* minimally invasive surgery

OS did not significantly differ between the MIS cohort and the open cohort {median OS 137 months (95% CI 107–not applicable [NA]) vs. 117 months (95% CI 103–NA), *p* = 0.77; HR 0.90 (95% CI 0.44–1.8), *p* = 0.77} (Fig. 1, left). Because the open cohort had more cases with a higher mesenteric mass grade, a higher tumor grade, and concurrent liver surgery, we used PSM to adjust for potential confounders. After PSM, the variables of both cohorts were well-balanced, with an SMD <0.2 or *p* value >0.3 (Online Resource Fig. 1, Online Resource Table 2), and the OS of the MIS cohort was still similar to that of the open surgery cohort (99 months [95% CI 91–NA] vs. 103 months [95% CI 86–NA], *p* = 0.77; HR 0.87 [95% CI 0.33–2.2], *p* = 0.77) (Fig. 1, right).

### Survival Analysis and Risk Factors for Conversion from Minimally Invasive Surgery to Open Surgery

**Fig. 2:**
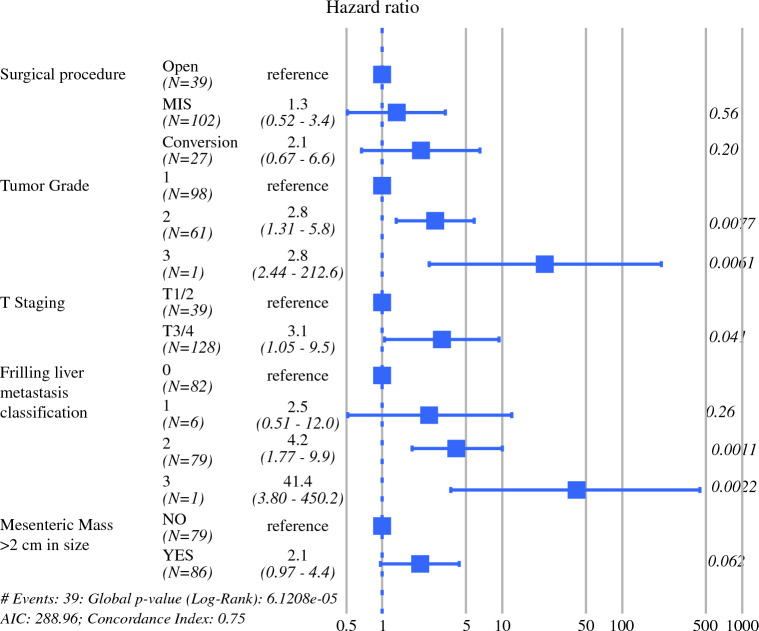
Multivariable analysis for overall mortality. The boxes represent hazard ratios for overall mortality and the lines represent 95% confidence intervals. *P* values are shown on the right. *MIS* minimally invasive surgery, *AIC* Akaike information criterion

We then focused on the varying mortality risks associated with the different surgical procedures, including conversion from MIS to open surgery, to detect possible effect modifiers and mediators associated with procedure indication. Multivariable analysis with known effect modifiers showed that the mortality risk of MIS conversion to open surgery (HR 2.1 [0.67–6.6], *p* = 0.20) and MIS without conversion were comparable with the open method (HR 1.3 [0.52–3.4], *p* = 0.56) (Fig. 2). Tumor grade (2, 3 vs. 1), T staging (3/4 vs. 1/2), Frilling liver metastasis classification (2, 3 vs. 0) and mesenteric mass >2 cm in size increased the risk of mortality (Fig. 2). Other than these known factors, we sought possible effect modifiers among the patient demographics and tumor characteristics between the open and conversion cohorts (Online Resource Table 3), and the only differentiating factor was race, which, in the open cohort, was White 76%, Asian 7.9%, Black 0%, Native American or Alaska Native 0%, other 16%, and in the conversion cohort was White 56%, Asian 0%, Black 19%, Native American or Alaska Native 3.7%, other 22% (*p* = 0.0072). As possible mediator factors, the occurrence of incomplete mesenteric resection, postoperative complications, and postoperative treatment were compared between the conversion cases and open surgery-alone cases, and there were no clear differences. Therefore, there was no clear evidence indicating worse survival in conversion from MIS to open surgery compared with open surgery.

## Discussion

Currently, the role of MIS for i-NETs has not been established.^3–5^ In this study, we compared the long-term OS of MIS using a hand-access port with a standard open surgery, finding no difference in OS after a median follow-up of more than 4 years. Since our study was retrospective and the disease characteristics of the two cohorts were not balanced, we performed PSM, which showed no difference in OS between MIS and open surgery. In contrast with our findings, a recent study by Kaçmaz et al. using the Netherlands Cancer Registry found that MIS was associated with improved OS compared with open surgery; however, detailed data on clinicopathological and radiographic features were lacking.^10^

As guidelines indicate, pure laparoscopic surgery is not adequate for identifying multiple primary tumors or resecting bulky mesenteric disease safely and completely.^3,4^ Some reports suggest that multifocality does not increase mortality and is not a contraindication for the laparoscopic method if the exteriorized bowel is palpated.^7^ The MIS procedure described here enables surgeons to investigate not only the whole jejunum-ileum but also the root of the mesentery via the hand-access port to facilitate oncological resection and safety.^9,11^ Tumoral invasion to the root of the mesentery is associated with incomplete resection.^9,20^ In these cases, palliative MIS may be done to alleviate obstruction or ischemia.^3–5^ In our study, the rates of complete mesenteric mass resection and morbidity were similar in the two PSM cohorts, which had similar rates of palliative surgery, hepatic metastasis, and mesenteric mass involvement. We selected open surgery when the mesenteric mass extended to the mesenteric root and a complete resection is deemed potentially possible. In some instances, when it is clear that complete mesenteric mass resection is not feasible, we performed a palliative MIS with care to preserve the superior mesenteric vessels during surgery.

In our study, multivariable analysis suggested that conversion from MIS to open surgery might have a potentially worse mortality risk, although there was no clear statistical evidence (Fig. 2). Conversion was performed in cases with bulky mesenteric lymphadenopathy or to perform a liver resection. Therefore, it was reasonable that the rate of hepatic metastasis and mesenteric mass extension to the mesenteric root were similar between the conversion and open cohorts (Online Resource Table 1). Our evaluation of mediator factors that might affect the mortality of the conversion and open cohorts showed no difference in completion of mesentery resection, morbidity, or postoperative treatments. Among patient charateristics, the only potential confounder was the low percentage of White patients and the high percentage of Black patients in the conversion cohort. Recent studies demonstrate different genomic backgrounds and prognoses in gastroenteropancreatic NETs by race.^21^ Pancreatic NETs of Black patients had a higher incidence of lymph node metastases in small primary tumors <2 cm in size, suggesting that a specific strategy might be needed for non-White patients.^22^ However, a post hoc Cox proportional hazards regression analysis showed no clear mortality risk for Black patients (HR 0.36 [0.085–1.5], *p* = 0.16), thus denying race as a clear effect modifier in our cohort. Since our multivariable analysis did not assume interactions in the model, other unknown different treatment effects could bias the results.

As there were no statistically proven differences in OS, we added analysis on progression-free survival (PFS) as a surrogate in a post hoc manner. PFS was defined as the length of time from the surgery date to disease progression, or death from any cause. PFS of the MIS cohort was still similar to that of the open surgery cohort in the entire cohort (45 months [95% CI 27–73] vs. 34 months [26–NA], *p* = 0.47; HR 0.83 [95% CI 0.50–1.4], *p* = 0.47) and in the PSM cohort (59 months [95% CI 36–NA] vs. 34 months [16–NA], *p* = 0.37; HR 0.74 [95% CI 0.38–1.4], *p* = 0.37) [Online Resource Fig. 2]. Furthermore, in multivariable analysis, there were no statistically proven differences in the progression risk of MIS conversion to open surgery (HR 1.4 [0.67–2.9], *p* = 0.38) and MIS without conversion was comparable with the open method (HR 0.98 [0.53–1.8], *p* = 0.94) [Online Resource Fig. 3]. Therefore, this analysis of PRS further supports our conclusion that MIS is an alternative to open surgery, ensuring similar survival outcomes.

As for non-survival outcomes, a lower frequency of postoperative complications (Grade ≥3) and shorter hospital stay for MIS methods were found in the entire cohort (Table 3), but not in the PSM cohort (Table 4). Prior studies reported shorter hospital stay for the laparoscopic method,^6,7^ but it should be noted that these researchers did not adjust other confounding variables by PSM or multivariable Cox regression analysis. On the other hand, MIS was associated with less intraoperative blood loss in the PSM cohort (Table 4), and this may be a potential advantage for patients who undergo MIS. Furthermore, MIS can achieve a similar mesenteric dissection margin with the open method (Table 4), therefore leading to comparable PFS and OS. This result was obtained with adjustment of the mesenteric mass status (see the Methods section, and Online resource Fig. 1), and thus excluded the selection bias of MIS cases. However, in clinical situations, we encounter severe mesenteric masses that have fibrosis, tethering, and thickening, but without involvement of the root of the mesentery. These conditions make resection more challenging. In these cases, particularly in patients with abundant visceral fat, open or conversion surgery may still be appropriate.

**Table 4:** Characteristics related to surgical outcomes of the propensity score-matched cohorts

Characteristic	*N*	Surgical procedure		
		Open [*n* = 31]	MIS [*n* = 55]	*p* value
Incomplete mesenteric lymph node dissection	86	6 (19)	10 (18)	1
Microscopically positive mesenteric margin	86	5 (16)	11 (20)	0.77
Complete mesenteric lymph node dissection	86	20 (65)	34 (62)	1
Estimated blood loss, mL [median (IQR)]	82	100 (35–150)	50 (20–100)	0.0071
Postoperative complications Grade ≥3	86	2 (6.5)	2 (3.6)	0.9
Length of stay, days [median (IQR)]	86	6 (5–7)	5 (4–7)	0.098
Postoperative treatment	86			
SSA or telotristat		13 (42)	26 (47)	0.66
Liver-directed therapy		13 (42)	26 (47)	0.66
Surgical resection		4 (13)	4 (7.3)	0.45
PRRT		6 (19)	10 (18)	1
Systemic medication		6 (19)	9 (16)	0.77
Survival status dead	86	7 (23)	13 (24)	1
Follow-up, months [median (IQR)]	86	46 (16–82)	53 (33–79)	0.31

Data are expressed as *n* (%) unless otherwise specified

*MIS* minimally invasive surgery, *IQR* interquartile range, *SSA* somatostatin analog, *PRRT* peptide receptor radionuclide therapy

This study has some limitations. First, as a single-institution, retrospective analysis, the study is subject to selection and referral bias. Since most surgeries for patients with i-NETs are performed minimally invasively at our institution, our findings require corroboration by studies elsewhere, given variable experience in advanced MIS worldwide.^23^ On the other hand, as a single-institution study of a large number of patients undergoing MIS for i-NETs, a major strength was the ability to capture granular data, such as clinicopathological features, that may not be present in large databases. Second, 108/168 patients included in our current study overlap with those in our previous publications on MIS for iNETs,^9,11^ and there remains the possibility of duplication or redundancy in the data. However, by reviewing the variables and the values, and by having a longer follow-up period and a larger cohort size in the current study than the prior studies that did not analyze OS, the risk of overstating the findings is thought to be minimalized. Third, with the smaller sample size after matching had been performed in order to better balance the potential confounders, the confidence limits around the HR estimate are fairly wide, but because they balance evenly above and below the null value of 1.0, it is very likely that a larger sample and more power would yield similar evidence that the effects on survival of the two surgical approaches of MIS and open surgery are neutral.

## Conclusion

MIS using a hand-access port is an alternative to open surgery for i-NETs, achieving similar short- and long-term oncological outcomes. Bulky mesenteric mass invading the mesenteric root and the need for concurrent liver resection are potential criteria for open surgery.

### Supplementary Information

Below is the link to the electronic supplementary material.

Supplementary file1 (DOCX 4224 kb)
