# Supplementary material for: Long-Term Survival Outcomes After Minimally Invasive Surgery for Ileal Neuroendocrine Tumors
Source: Ann Surg Oncol. 2024 May 26;31(9):5507–14. doi: 10.1245/s10434-024-15468-6 (PMC11300575; doi:10.1245/s10434-024-15468-6)
Supplement: Supplementary file 1 — Supplementary file1 (DOCX 4224 kb) [file 10434_2024_15468_MOESM1_ESM.docx]

**List of Supporting Information:**

**Supplementary Table 1. Demographics and clinical characteristics of the entire cohorts by open surgery, MIS** **without conversion, and conversion.**

**Supplementary Table 2. Demographics and clinical characteristics of the propensity-score-matched cohorts.**

**Supplementary Table 3. Demographics and clinical characteristics and tumor characteristics of the open and conversion cohort.**

**Supplementary Fig 1. Adjusted Standardized Mean Differences by Propensity-Score Matching.**

**Supplementary Fig 2. Progression-free survival curves.**

**Supplementary Fig 3. Multivariable analysis for disease progression.**

**Supplementary Table 1. Demographics and clinical characteristics of the entire cohorts by open surgery, MIS** **without conversion, and conversion.**

|  | | **Surgical Procedure, N(%)** | | |  |
| --- | --- | --- | --- | --- | --- |
| **Characteristic** | **N** | **Open**, N = 39 | **MIS**, N = 102 | **Conversion**, N = 27 | **p-value** |
| **Age, Median (IQR)** | 168 | 61 (53, 67) | 60 (52, 66) | 59 (54, 69) | 0.68^1^ |
| **Gender Female** | 168 | 20 (51) | 53 (52) | 9 (33) | 0.2 |
| **BMI (kg/m^2^) , Median (IQR)** | 167 | 27.0 (24.2, 29) | 25.2 (22.5, 31) | 29.2 (25.4, 31) | 0.24^1^ |
| **Race White** | 165 | 29 (76) | 82 (82) | 15 (56) | 0.016 |
| **Carcinoid syndrome** | 165 | 20 (53) | 51 (51) | 18 (67) | 0.3 |
| **SSA before resection** | 168 | 14 (36) | 28 (27) | 10 (37) | 0.5 |
| **History of small bowel obstruction** | 168 | 10 (26) | 31 (30) | 10 (37) | 0.6 |
| **Size of primary tumor (cm) , Median (IQR)** | 166 | 1.9 (1.3, 3) | 2.0 (1.3, 3) | 1.8 (1.4, 3) | 0.89* |
| **Number of primary tumor(s) , Median (IQR)** | 167 | 1.0 (1.0, 4) | 1.0 (1.0, 2) | 1.0 (1.0, 4) | 0.46^1^ |
| **Multiple tumors** | 167 | 17 (45) | 36 (35) | 13 (48) | 0.4 |
| **Mesenteric mass involvement to the root of the mesentery** | 168 | 9 (23) | 3 (2.9) | 10 (37) | <0.001 |
| **Size of mesenteric mass (cm), Median (IQR)** | 165 | 2.6 (1.1, 3.5) | 1.5 (1.0, 2.9) | 2.5 (1.6, 4.4) | 0.0034^1^ |
| **Mesenteric mass > 2 cm in size** | 165 | 25 (66) | 44 (44) | 17 (63) | 0.034 |
| **Tumor Grade** | 160 |  |  |  | 0.3 |
| 1 |  | 18 (49) | 64 (65) | 16 (64) |  |
| 2 |  | 19 (51) | 33 (34) | 9 (36) |  |
| 3 |  | 0 (0) | 1 (1.0) | 0 (0) |  |
| **T Staging** | 167 |  |  |  | 0.2 |
| T1 |  | 2 (5.3) | 8 (7.8) | 2 (7.4) |  |
| T2 |  | 3 (7.9) | 23 (23) | 1 (3.7) |  |
| T3 |  | 19 (50) | 45 (44) | 15 (56) |  |
| T4 |  | 14 (37) | 26 (25) | 9 (33) |  |
| **N +** | 164 | 36 (95) | 98 (97) | 24 (96) | 0.7 |
| **M Staging** | 168 |  |  |  | 0.5 |
| M0 |  | 13 (33) | 49 (48) | 10 (37) |  |
| M1a |  | 13 (33) | 33 (32) | 12 (44) |  |
| M1b |  | 4 (10) | 5 (4.9) | 1 (3.7) |  |
| M1c |  | 9 (23) | 15 (15) | 4 (15) |  |
| **Stage** | 168 |  |  |  | 0.4 |
| I |  | 0 (0) | 3 (2.9) | 1 (3.7) |  |
| II |  | 1 (2.6) | 1 (1.0) | 0 (0) |  |
| III |  | 12 (31) | 45 (44) | 9 (33) |  |
| IV |  | 26 (67) | 53 (52) | 17 (63) |  |
| **Frilling Liver Metastasis Classification** | 168 |  |  |  | 0.027 |
| 0 |  | 17 (44) | 54 (53) | 11 (41) |  |
| 1 |  | 1 (2.6) | 1 (1.0) | 4 (15) |  |
| 2 |  | 20 (51) | 47 (46) | 12 (44) |  |
| 3 |  | 1 (2.6) | 0 (0) | 0 (0) |  |
| **Concurrent liver surgery** | 168 | 10 (26) | 3 (2.9) | 6 (22) | <0.001 |
| **Incomplete mesenteric lymph node dissection** | 167 | 9 (23) | 7 (6.9) | 11 (41) | <0.001 |
| **Microscopically positive mesenteric margin** | 168 | 7 (18) | 16 (16) | 4 (15) | >0.9 |
| **Complete mesenteric lymph node dissection** | 167 | 23 (59) | 78 (77) | 12 (44) | 0.002 |
| **Estimated blood loss (ml), median (IQR)** | 161 | 100 (30,150) | 50 (10, 50) | 100 (50, 100) | 0.0068^1^ |
| **Postoperative　complications >= G3** | 168 | 4 (10) | 4 (3.9) | 0 (0) | 0.13 |
|  |  |  |  |  |  |
| **Length of stay (days), Median (IQR)** | 168 | 6 (5,7) | 5 (4,6) | 7 (6,8) | 0.16^1^ |
| **Postoperative treatment** |  |  |  |  |  |
| SSA or telotristat | 168 | 19 (49) | 43 (42) | 18 (67) | 0.076 |
| Liver directed therapy | 168 | 10 (26) | 26 (25) | 4 (15) | 0.5 |
| Surgical resection | 168 | 5 (13) | 9 (8.8) | 4 (15) | 0.6 |
| PRRT | 168 | 6 (15) | 16 (16) | 3 (11) | >0.9 |
| Systemic medication | 168 | 8 (21) | 11 (11) | 3 (11) | 0.4 |
| **Survival status Dead** | 167 | 10 (26) | 23 (23) | 11 (41) | 0.2 |
| **Follow-up months, Median (IQR)** | 168 | 46 (16, 99) | 49 (26, 90) | 55 (23, 74) | 0.63^1^ |

BMI, Body mass index; SSA, Somatostatin analogue; ^1^ANOVA test

**Supplementary Table 2. Demographics and clinical characteristics of the propensity-score-matched cohorts.**

|  | | **Surgical Procedure, N(%)** | |  |
| --- | --- | --- | --- | --- |
| **Characteristic** | **N** | **Open**, N = 31 | **MIS**, N = 55 | **p-value** |
| **Surgical Procedure** | 86 |  |  |  |
| Open |  | 31 (100) | 0 (0) |  |
| MIS without conversion |  | 0 (0) | 43 (78) |  |
| MIS converted to Open |  | 0 (0) | 12 (22) |  |
| **Age** | 86 | 61 (53, 68) | 62 (56, 68) | 0.90 |
| **Gender Female** | 86 | 15 (48) | 30 (55) | 0.66 |
| **BMI (kg/m^2^)** | 86 | 27.1 (24.2, 29) | 25.4 (22.2, 31) | 0.65 |
| **Race White** | 86 | 24 (77) | 45 (82) | 0.78 |
| **Carcinoid syndrome** | 84 | 14 (47) | 24 (44) | 1 |
| **SSA before resection** | 86 | 11 (35) | 13 (24) | 0.32 |
| **History of small bowel obstruction** | 86 | 8 (26) | 13 (24) | 1 |
| **Size of primary tumor (cm), Median (IQR)** | 85 | 2.0 (1.4, 3) | 1.8 (1.5, 3) | 0.82 |
| **Number of primary tumor(s), Median (IQR)** | 86 | 1.0 (1.0, 4) | 1.0 (1.0, 3) | 0.84 |
| **Multiple tumor** | 86 | 13 (42) | 26 (47) | 0.66 |
| **Mesenteric mass involvement to the root of the mesentery** | 86 | 5 (16) | 8 (15) | 1 |
| **Size of mesenteric mass (cm), Median (IQR)** | 86 | 2.40 (1.05, 3.55) | 2.50 (1.40, 3.85) | 0.48 |
| **Mesenteric mass > 2 cm in size** | 86 | 20 (65) | 38 (69) | 0.81 |
| **Tumor Grade** | 86 |  |  | 1 |
| 1 |  | 17 (55) | 30 (55) |  |
| 2 |  | 14 (45) | 25 (45) |  |
| 3 |  | 0 (0) | 0 (0) |  |
| **T Staging** | 86 |  |  | 0.68 |
| T1 |  | 1 (3.2) | 3 (5.5) |  |
| T2 |  | 1 (3.2) | 3 (5.5) |  |
| T3 |  | 16 (52) | 33 (60) |  |
| T4 |  | 13 (42) | 16 (29) |  |
| **N +** | 86 | 1 (3.2) | 2 (3.6) | 1 |
| **M Staging** | 86 |  |  | 0.46 |
| M0 |  | 12 (39) | 24 (44) |  |
| M1a |  | 9 (29) | 21 (38) |  |
| M1b |  | 3 (9.7) | 4 (7.3) |  |
| M1c |  | 7 (23) | 6 (11) |  |
| **Stage** | 86 |  |  | 0.49 |
| I |  | 0 (0) | 2 (3.6) |  |
| II |  | 1 (3.2) | 0 (0) |  |
| III |  | 11 (35) | 22 (40) |  |
| IV |  | 19 (61) | 31 (56) |  |
| **Frilling Liver Metastasis Classification** | 86 |  |  | 0.68 |
| 0 |  | 15 (48) | 28 (51) |  |
| 1 |  | 1 (3.2) | 3 (5.5) |  |
| 2 |  | 14 (45) | 24 (44) |  |
| 3 |  | 1 (3.2) | 0 (0) |  |
| **Concurrent liver surgery** | 86 | 6 (19) | 8 (15) | 0.6 |
| BMI, Body mass index; SSA, Somatostatin analogue | | | | |

**Supplementary Table 3. Demographics and clinical characteristics and tumor characteristics of the open and conversion cohort.**

|  | | **Surgical Procedure, N(%)** | |  |
| --- | --- | --- | --- | --- |
| **Characteristic** | **N** | **Open**, N = 39 | **conversion**, N = 27 | **p-value** |
| **Age** | 66 | 61 (53, 67) | 59 (54, 69) | 0.95 |
| **Gender Female** | 66 | 20 (51%) | 9 (33%) | 0.21 |
| **BMI (kg/m^2^)** | 66 | 27.0 (24.2, 29) | 29.2 (25.4, 31) | 0.11 |
| **Race White** | 65 | 29 (76%) | 15 (56%) | 0.10 |
| **Race in detail** | 65 |  |  | 0.0072 |
| Asian |  | 3 (7.9%) | 0 (0%) |  |
| Black or African American |  | 0 (0%) | 5 (19%) |  |
| Native American or Alaska Native |  | 0 (0%) | 1 (3.7%) |  |
| Other |  | 6 (16%) | 6 (22%) |  |
| White |  | 29 (76%) | 15 (56%) |  |
| **Carcinoid syndrome** | 65 | 20 (53%) | 18 (67%) | 0.61 |
| **SSA before resection** | 86 | 14 (36%) | 10 (37%) | 1 |
| **History of small bowel obstruction** | 66 | 10 (26%) | 10 (37%) | 0.41 |
| **Size of primary tumor (cm), Median (IQR)** | 64 | 1.9 (1.3, 3) | 1.8 (1.4, 3) | 0.81 |
| **Number of primary tumor(s), Median (IQR)** | 64 | 1.0 (1.0, 4) | 1.0 (1.0, 4) | 0.90 |
| **Multiple tumor** | 65 | 17 (45%) | 13 (48%) | 0.81 |
| **Mesenteric mass involvement to the root of the mesentery** | 66 | 9 (23%) | 10 (37%) | 0.27 |
| **Size of mesenteric mass (cm), Median (IQR)** | 65 | 2.60 (1.13, 3.50) | 2.50 (1.60, 4.35) | 0.43 |
| **Mesenteric mass > 2 cm in size** | 65 | 25 (66%) | 17 (63%) | 1 |
| **Tumor Grade** | 62 |  |  | 0.30 |
| 1 |  | 18 (49%) | 16 (64%) |  |
| 2 |  | 19 (51%) | 9 (36%) |  |
| 3 |  | 0 (0%) | 0 (0%) |  |
| **T Staging** | 65 |  |  | 0.90 |
| T1 |  | 2 (5.3%) | 2 (7.4%) |  |
| T2 |  | 3 (7.9%) | 1 (3.7%) |  |
| T3 |  | 19 (50%) | 15 (56%) |  |
| T4 |  | 14 (37%) | 9 (33%) |  |
| **N +** | 63 | 36 (95%) | 24 (96%) | 1 |
| **M Staging** | 66 |  |  | 0.62 |
| M0 |  | 13 (33%) | 10 (37%) |  |
| M1a |  | 13 (33%) | 12 (44%) |  |
| M1b |  | 4 (10%) | 1 (3.7%) |  |
| M1c |  | 9 (23%) | 4 (15%) |  |
| **Stage** | 66 |  |  | 0.80 |
| I |  | 0 (0%) | 1 (3.7%) |  |
| II |  | 1 (2.6%) | 0 (0%) |  |
| III |  | 12 (31%) | 9 (33%) |  |
| IV |  | 26 (67%) | 17 (63%) |  |
| **Frilling Liver Metastasis Classification** | 66 |  |  | 0.28 |
| 0 |  | 17 (44%) | 11 (41%) |  |
| 1 |  | 1 (2.6%) | 4 (15%) |  |
| 2 |  | 20 (51%) | 12 (44%) |  |
| 3 |  | 1 (2.6%) | 0 (0%) |  |
| **Concurrent liver surgery** | 66 | 10 (26%) | 6 (22%) | 1 |
| BMI, Body mass index; SSA, Somatostatin analogue | | | | |

**Supplementary Fig 1. Adjusted Standardized Mean Differences by Propensity-Score Matching.** Standardized mean differences of the variables are represented by red and green dots in unmatched and matched cohorts respectively.


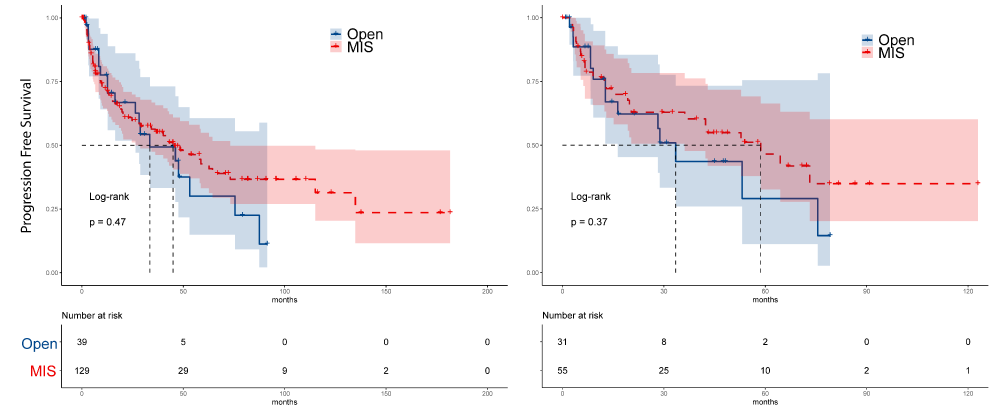


**Supplementary Fig 2. Progression-free survival curves.** Kaplan-Meier progression-free survival curves are shown for the entire cohort (left) and the propensity-score-matched cohorts (right). Shading areas are 95% confidence intervals.

**
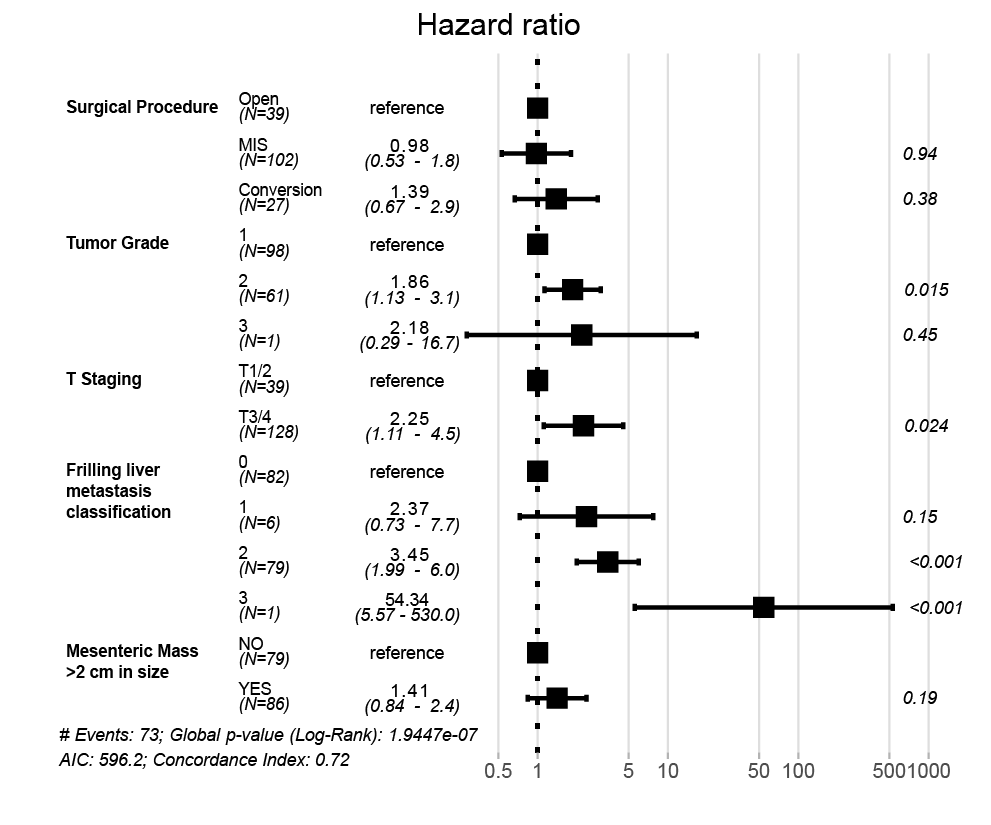
**

**Supplementary Fig 3. Multivariable analysis for disease progression.** The boxes represent hazard ratios for disease progression and the lines represent 95% confidence intervals. P-values are shown on the ri
